# Magnitude of A1C improvement in relation to baseline A1C and amount of weight loss in response to intensive lifestyle intervention in real‐world diabetes practice: 13 years of observation

**DOI:** 10.1111/1753-0407.13395

**Published:** 2023-05-16

**Authors:** Ahmed H. Eldib, Shilton Dhaver, Marwa Al‐Badri, Tareq Salah, Karim Kibaa, Omnia Elenani, Shaheen Tomah, Hannah Gardner, Osama Hamdy

**Affiliations:** ^1^ Joslin Diabetes Center Boston Massachusetts USA; ^2^ Harvard Medical School Boston Massachusetts USA

**Keywords:** A1C reduction, diabetes, glycemic control, intensive lifestyle intervention, weight loss, 糖尿病, HbA_1C_降低, 血糖控制, 强化生活方式干预, 减重

## Abstract

**Background:**

Effect of intensive lifestyle intervention (ILI) on A1C in participants with diabetes is underestimated. A1C improvement is presumed to be dependent on the amount of weight loss. Here, we evaluate the magnitude of A1C change in relation to baseline A1C and the amount of weight loss in participants with diabetes who underwent ILI over 13 years in real‐world clinical practice.

**Methods:**

A total of 590 participants with diabetes were enrolled in the Weight Achievement and Intensive Treatment (Why WAIT) program, a 12‐week multidisciplinary ILI program designed for real‐world clinical practice between September 2005 and May 2018. We stratified participants based on baseline A1C into three groups: group A: A1C ≥ 9%, group B: A1C 8 to <9%, and group C: A1C ≥6.5% to <8%.

**Results:**

After 12‐weeks of intervention, body weight decreased in all groups, and pairwise comparisons of A1C changes showed that: group A had 1.3% greater A1C reduction than group B (*p* = 0.0001) and 2% greater than group C (*p* = 0.0001), while group B had 0.7% greater A1C reduction than group C (*p* = 0.0001).

**Conclusion:**

We conclude that ILI may decrease A1C by up to 2.5% in participants with diabetes. At similar magnitude of weight loss, A1C reduction was more prominent in participants with higher baseline A1C. This may be valuable for clinicians to set a realistic expectation of A1C change in response to ILI.

## Graphical abstract

**Highlights**

- Intensive lifestyle intervention decreases A1C and body weight in patients with diabetes by up to 2.5%. At similar magnitude of weight loss, A1C reduction is more prominent in participants with higher baseline A1C.

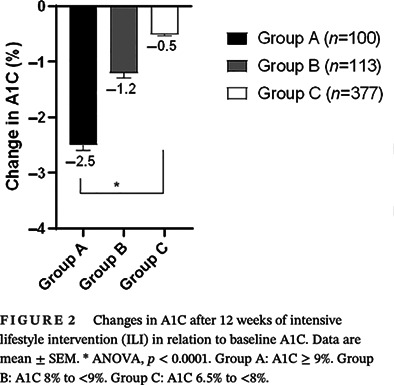

## 摘要

**【摘要】 背景** 强化生活方式干预(ILI)对于糖尿病患者的HbA_1C_效果被低估。HbA_1C_ 的改善被认为依赖于体重的减少程度。在真实世界的临床实践中，本研究评估了在经历13年以上强化生活方式干预的糖尿病患者中，与基线HbA_1C_ 相关的HbA_1C_ 变化幅度与体重减轻程度。**方法** 从2005年9月至2018年5月，在真实世界的临床实践中，共有590例糖尿病患者参加了体重达标和强化治疗(Why WAIT)项目，这是1个为期12周的多学科ILI项目。本研究根据基线HbA_1C_将参与者分为3组:A组:HbA_1C_ >9%，B组:HbA_1C_ 8%~9%，C组:HbA_1C_ 6.5%~8%。**结果** 经过12周的干预后，所有组患者的体重均有所下降，HbA_1C_变化的两两比较显示:A组的HbA_1C_降低幅度比B组高1.3%(*P*=0.0001)，比C组高2%(*P*=0.0001)，而B组的HbA_1C_降低幅度比C组高0.7%(*P*=0.0001)。**结论** 本研究提示，ILI可能使糖尿病患者的HbA_1C_降低高达2.5%。在相似的体重减轻幅度下，基线HbA_1C_较高的参与者HbA_1C_降低更为明显。这对临床医生来说，设置与ILI相应的HbA_1C_变化的现实预期可能是有价值的。

## 1 INTRODUCTION

The effect of intensive lifestyle intervention (ILI) on A1C in real‐world clinical practice is inconsistent and frequently underestimated.^1^ As per diabetes management guidelines, physicians decide on the number and type of initial anti‐hyperglycemic medications based on baseline A1C.^2^ The higher the baseline A1C, the more medications are recommended to rapidly lower A1C. Recent data indicate that only 56% of patients with diabetes were asked to follow a physical exercise program by their providers.^3^ Anderson and Moore suggest that poorly controlled hyperglycemia in participants with type 2 diabetes (T2D) could be improved by devoting time during office visits to discuss lifestyle modifications.^4^ It was also presumed that the magnitude of A1C improvement and changes in cardiovascular risk factors are solely dependent on the magnitude of weight loss.^5^, ^6^ It is not clear the magnitude of A1C change compared in relation to baseline A1C and the amount of weight loss in participants with diabetes who enroll in intensive lifestyle intervention (ILI) in real‐world clinical practice. Therefore, we conducted this retrospective analysis of a large cohort of participants with diabetes and obesity, who enrolled in a 12‐week multidisciplinary ILI over 13 years in real‐world clinical practice. We evaluated the magnitude of changes in A1C, body weight, and other cardiovascular risk factors in relation to baseline A1C and amount of weight loss.

## 2 METHODS

### 2.1 The Why WAIT program

Weight Achievement and Intensive Treatment (Why WAIT) is a 12‐week multidisciplinary ILI program designed for weight reduction and intensive diabetes management in real‐world clinical practice. The program started in 2005 at Joslin Diabetes Center in Boston, MA. Full descriptions of the program have been published elsewhere.^7^ After clinical evaluation by a multidisciplinary team of endocrinologists, dietitians, exercise physiologists and psychologists, participants with either type 1 diabetes (T1D) or type 2 diabetes (T2D) and with a body mass index (BMI) between 30 and 45 kg/m^2^ are enrolled in the program. Why WAIT program includes: group education, dietary intervention, medications' adjustment, exercise intervention and cognitive‐behavioral modifications. This study was approved by the Committee on Human Studies (CHS) at the Joslin Diabetes Center. We retrospectively reviewed the baseline and 12‐week electronic medical records of 590 participants with diabetes and obesity, who enrolled in the Why WAIT program between September 2005 and May 2018.

### 2.2 Statistical analysis

Demographic and baseline characteristics were evaluated using descriptive statistics and Chi square test. Continuous variables are reported as means ± standard deviations (SD) or standard error of the mean (SEM). Categorical variables were presented as numbers or percentages. Each of the reported variables was tested for having a significant change from the baseline after the intervention using paired *t*‐test. ANOVA was used to compare the difference of the mean change for each of the variables between the three groups. Linear regression was eventually used to account for potential confounders. Statistical significance was defined as two‐tail *p* values <0.05. Analysis was conducted using StataSE‐64 version (StataCorp®, College Station, Texas, USA 2017).

## 3 RESULTS

**FIGURE 1:**
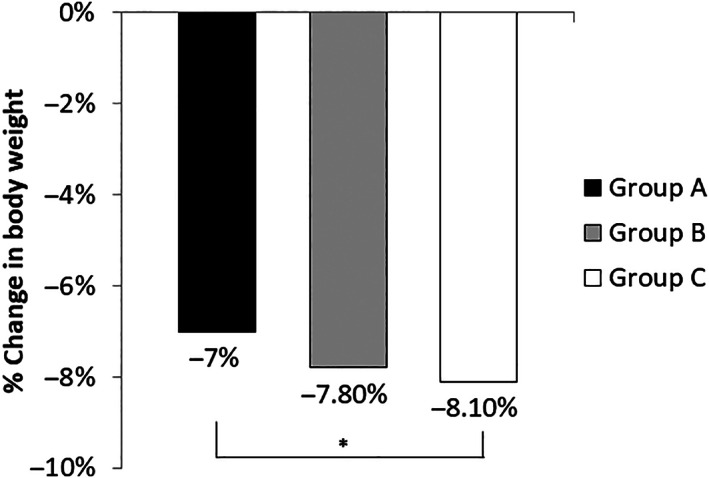
Changes in body weight after 12 weeks of intensive lifestyle intervention (ILI) in relation to baseline A1C. Data are mean + SEM. * ANOVA, *p* = 0.22. Group A: A1C ≥ 9%. Group B: A1C 8% to <9%. Group C: A1C 6.5% to <8%.

**FIGURE 2:**
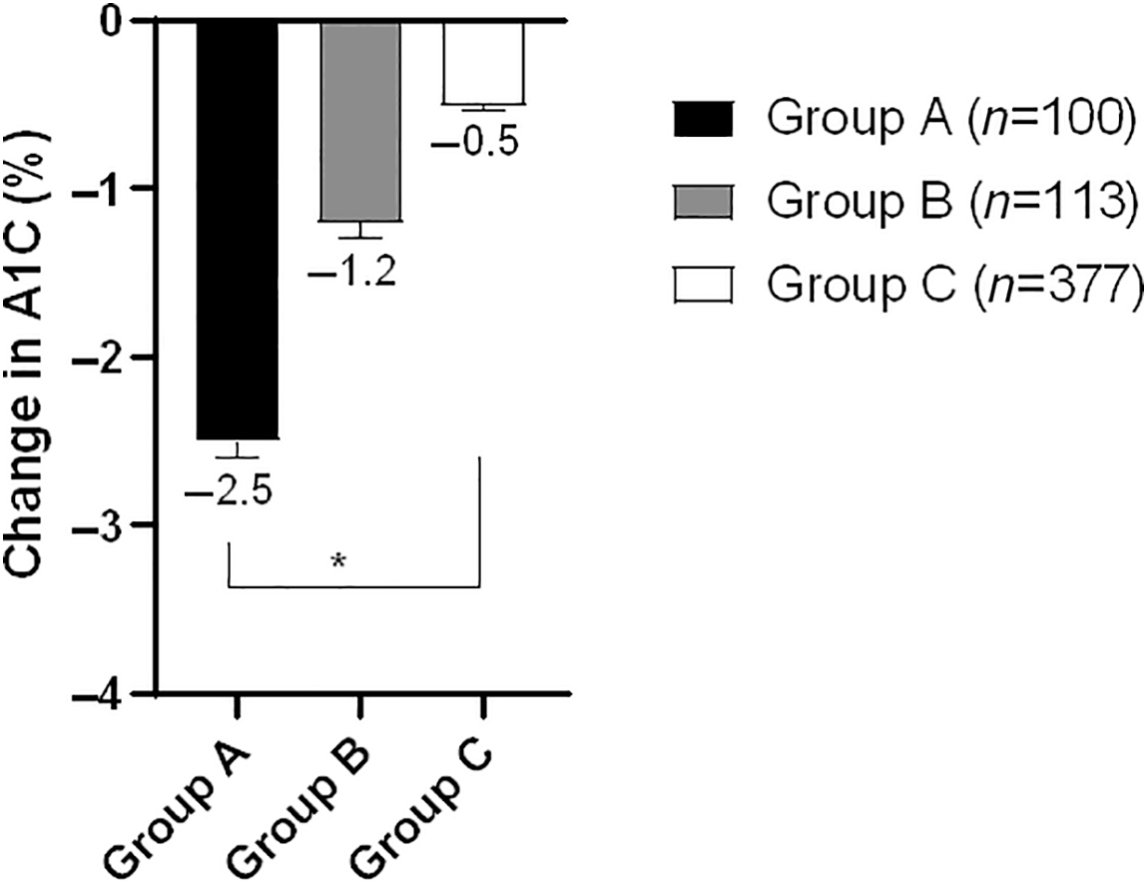
Changes in A1C after 12 weeks of intensive lifestyle intervention (ILI) in relation to baseline A1C. Data are mean ± SEM. * ANOVA, *p* < 0.0001. Group A: A1C ≥ 9%. Group B: A1C 8% to <9%. Group C: A1C 6.5% to <8%.

**FIGURE 3:**
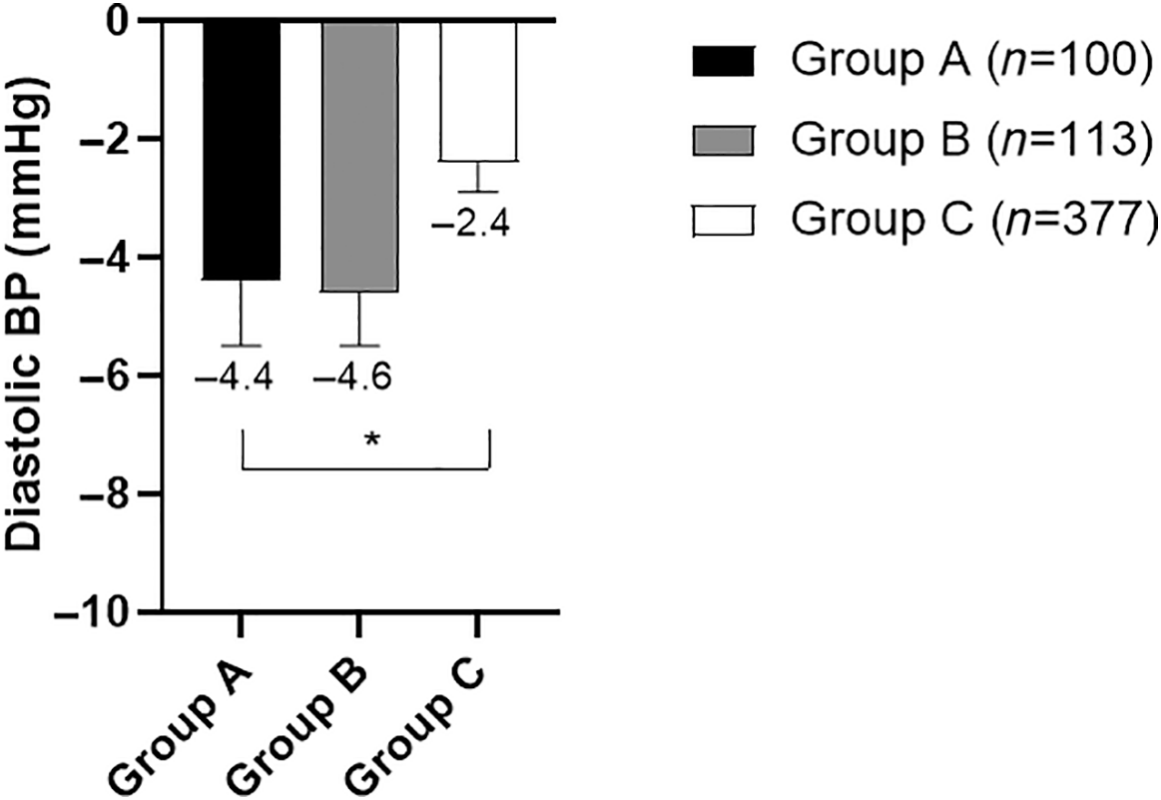
Changes in diastolic blood pressure after 12 weeks of intensive lifestyle intervention (ILI) in relation to baseline A1C. Data are mean + SEM. * ANOVA, *p* = 0.03. Group A: A1C ≥ 9%. Group B: A1C 8% to <9%. Group C: A1C 6.5% to <8%.

**FIGURE 4:**
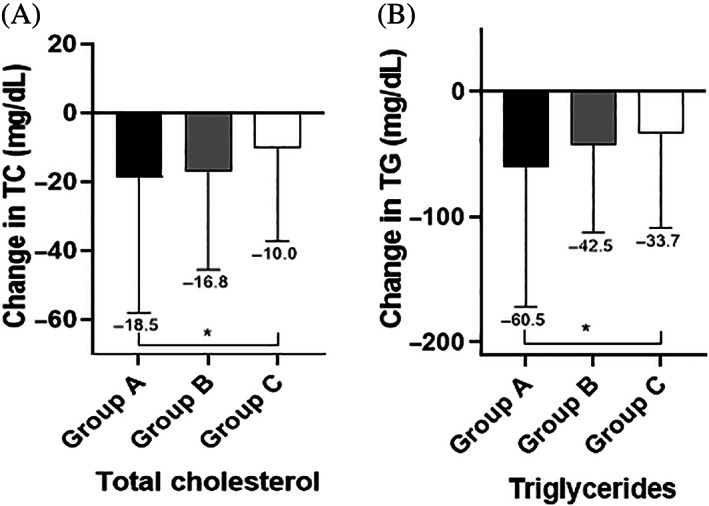
Changes in total cholesterol (A) and triglycerides (B) after 12 weeks of ILI in relation to baseline A1C. Data are mean ± SEM. * ANOVA, *p* = 0.02. **ANOVA, *p* = 0.02. Group A: A1C ≥ 9%. Group B: A1C 8% to <9%. Group C: A1C 6.5% to <8%.

The study included 590 participants (age 52.5 ± 11 years, T1D 16.3% and females 58.6%). According to baseline A1C, we stratified participants into three groups: Group A with A1C ≥9% (16.95%), Group B with A1C 8% to <9% (19.15%), Group C with A1C ≥6.5% to <8% (63.9%). At baseline, there were no significant differences between the three study groups in gender, body weight, BMI, LDL‐cholesterol, and HDL‐cholesterol. There were significant differences in age, percentage of participants with T1D, diabetes duration, systolic BP, diastolic BP, total cholesterol (TC), triglycerides (TG), percentage of participants treated with insulin. (Table 1). We longitudinally evaluated changes in body weight, BMI, A1C, blood pressure (BP), and lipid profile after 12‐weeks of ILI (Table 2). All groups achieved significant weight reduction after 12‐weeks of ILI. The amount of weight reduction did not differ between the three groups (*p* = 0.22) (Figure 1). They also achieved similar improvement in BMI (*p*‐0.7). These findings did not change after adjusting for age, sex, diabetes type, diabetes duration, and percentage of participants treated with insulin. All groups achieved significant A1C reduction after 12‐weeks of ILI (Figure 2). The amount of A1C reduction differed significantly between the three groups (*p* < 0.0001). Upon performing Bonferroni correction, group A achieved a significantly greater A1C reduction than group B (−1.3%, *p* < 0.001) and group C (−2.0%, *p* < 0.001). Additionally, group B had greater A1C reduction than group C (−0.7%, *p* < 0.001). The difference in A1C reduction between the three groups remained statistically significant after adjusting for age, sex, diabetes type, diabetes duration, and percentage of participants treated with insulin (*p* < 0.001). All groups achieved significant reduction in systolic BP and diastolic BP after 12‐weeks of ILI (Figure 3). Groups A and B seemed to have better reduction in diastolic BP than group C (*p* = 0.03). Reduction in systolic blood pressure was not significantly different between the three groups (*p* = 0.06), however after adjusting for age, sex, diabetes type, and diabetes duration, the reduction in systolic BP was significantly different between the three groups, being the highest in group A (*p* = 0.02). The three groups showed significant decrease in TC, LDL‐cholesterol, and TG after 12‐weeks of ILI (Figure 4). HDL‐cholesterol did not change in any of the three groups. Groups A and B showed more prominent reductions in TC than group C (*p* = 0.02), but the difference disappeared after adjusting for age, sex, diabetes type, diabetes duration and insulin therapy (*p* = 0.15). Group A showed greater reduction in TG than groups B and C (*p* = 0.02). There were no significant differences between the three groups in the amount of LDL‐cholesterol reduction (0.31). The TG, LDL‐cholesterol and HDL‐cholesterol findings remained the same after adjustment for age, sex, diabetes type, diabetes duration, and percentage of participants on insulin.

**TABLE 1:** Baseline characteristics of participants with diabetes and obesity enrolled in 12 weeks of ILI.

Variable	Total cohort (*N* = 590)	Group A (*n* = 100) A1C ≥9%	Group B (*n* = 113) A1C 8–<9%	Group C (*n* = 377) A1C 6.5–<8%	*p* value^a^
Female *n* (%)	346 (58.6%)	52 (52%)	66 (58.4%)	228 (60.5%)	0.3
Diabetes type 1 *n* (%)	96 (16.3%)	23 (23%)	36 (31.9%)	37 (9.8%)	<0.001
Age (years)	52.5 ± 11.0	51.0 ± 10.9	50.4 ± 11.5	53.5 ± 10.8	0.01
Body weight (kg)	105.5 ± 18.6	108.4 ± 20.2	102.9 ± 16.5	105.5 ± 18.7	0.1
BMI (kg/m^2^)	36.7 ± 5.1	36.7 ± 5.1	36.0 ± 4.8	36.9 ± 5.2	0.3
diabetes duration (years)	10.9 ± 9.8	14.1 ± 10.3	14.5 ± 11.4	9.0 ± 8.6	<0.001
A1C (%)	7.7 ± 1.4	9.9 ± 0.9	8.4 ± 0.3	6.9 ± 0.6	<0.001
Systolic BP (mmHg)	128.9 ± 15.7	133.1 ± 16.9	130.7 ± 16.4	127.4 ± 15.0	0.004
Diastolic BP (mmHg)	77.6 ± 9.1	79.7 ± 8.9	77.8 ± 9	76.9 ± 9.1	0.04
Total cholesterol (mg/dL)	166.2 ± 35.1	174.3 ± 43.7	168.3 ± 34.6	163.6 ± 32.5	0.03
LDL‐cholesterol (mg/dL)	95.9 ± 29.9	97.8 ± 34.2	99.1 ± 28.7	94.4 ± 29.2	0.3
HDL‐cholesterol (mg/dL)	47.5 ± 14.5	46.6 ± 14.6	48.5 ± 16.7	47.5 ± 13.8	0.6
Triglycerides (mg/dL)	153.9 ± 105.4	181.7 ± 174.0	151.6 ± 91.5	147.5 ± 83.3	0.02
Insulin treatment *n* (%)	292 (49.8%)	78 (78.8%)	80 (71.4%)	134 (35.7%)	<0.001

*Note*: Values are mean ± SD.

Abbreviations: BP, blood pressure; HDL, high‐density lipoprotein; ILI, intensive lifestyle intervention; LDL, low‐density lipoprotein.

^a^ One‐way ANOVA or Chi square.

**TABLE 2:** Changes in body weight, A1C, lipid profile and blood pressure in participants with diabetes and obesity after 12 weeks of ILI in relation to baseline A1C.

Variable	Group A (*n* = 100) A1C ≥ 9%	Group B (*n* = 113) A1C 8% to <9%	Group C (*n* = 377) A1C 6.5% to <8%	*p* value^a^
Body weight (Kg)	−−7.6 ± 0.6*	−8.0 ± 0.4*	−8.5 ± 0.2*	0.22
BMI (Kg/m^2^)	−2.2 ± 0.3*	−2.8 ± 0.2*	−1.3 ± 1.1	0.7
A1C (%)	−2.5 ± 0.1*	−1.2 ± 0.1*	−0.5 ± 0.03*	<0.0001
Systolic BP (mmHg)	−7.7 ± 2.0*	−9.9 ± 1.5*	−5.8 ± 0.8*	0.06
Diastolic BP (mmHg)	−4.4 ± 1.1*	−4.6 ± 0.9*	−2.4 ± 0.5*	0.03
Total cholesterol (mg/dL)	−18.5 ± 39.5*	−16.8 ± 28.7*	−10.0 ± 27.2*	0.02
LDL‐cholesterol (mg/dL)	−11.7 ± 24.5*	−11.2 ± 24.6*	−8.0 ± 23.2*	0.31
HDL‐cholesterol (mg/dL)	0.5 ± 8.5	−1.2 ± 7.1	0.5 ± 8.2	0.16
Triglycerides (mg/dL)	−60.5 ± 111.3*	−42.5 ± 70.1*	−33.7 ± 75.2*	0.02

*Note*: Values are mean ± SE.

Abbreviations: BP, blood pressure; HDL, high‐density lipoprotein; ILI, intensive lifestyle intervention; LDL, low‐density lipoprotein.

^a^ One‐way ANOVA. * significant change from baseline using paired *t*‐test.

We also stratified participants based on diabetes type and performed linear regression analysis with adjustment for age, sex, diabetes duration, and percentage of participants on insulin to see if the study findings would change in each subgroup. Both T1D and T2D participants showed similar patterns of increased magnitude of A1C reduction in association with the higher baseline A1C (T1D cohort: *r* ^2^ = 0.36, *p* < 0.001, T2D cohort: *r* ^2^ = 0.56, *p* < 0.001) (Table 3).

**TABLE 3:** Association of change in systolic BP and change in total cholesterol, and baseline A1C.

	Unadjusted model		Adjusted model	
	*β* (95% CI)	*p* value^a^	*β* (95% CI)	*p* value^a^
Change in systolic BP after 12 weeks of ILI	−1.46 (−3.23 to 0.32)	0.11	−2.30 (−4.25 to −0.34)	0.02
Change in Total cholesterol after 12 weeks of ILI	−4.68 (−7.99 to −1.36)	0.006	−2.64 (−6.25 to 0.98)	0.15

*Note*: Adjusted model for age, sex, diabetes type, and diabetes duration.

Abbreviation: BP, blood pressure; ILI, intensive lifestyle intervention.

^a^ Linear regression.

## 4 DISCUSSION

We previously showed that A1C decreased significantly by an average of 1.0% (−1.5 to −0.4%) after 12 weeks of ILI, and that participants in Why WAIT program were able to maintain weight loss of 6.4% after 5 years.^8^, ^9^, ^10^

In this study, we stratified participants according their baseline A1C. All participants achieved significant weight reduction and improvement in A1C, however A1C reduction was more prominent among participants with higher baseline A1C despite achieving similar magnitude of weight reduction. All participants also had significant improvement in BP and lipid profile.

Our findings showed that multidisciplinary ILI for 12‐weeks in real‐world clinical practice decreased body weight by an average of 7.9%, and decreased A1C by up to 2.5%. Having a large cohort to evaluate the impact of baseline A1C on their glycemic response to the ILI.

The three groups had similar baseline body weight and they lost similar weight after 12‐weeks of ILI. Participants with the highest baseline A1C (group A) achieved a mean A1C reduction of (−2.5% ± 0.1%), while participants with the modestly high baseline A1C (group B) achieved a mean A1C reduction of −1.2% ± 0.1% and participants with the lowest baseline A1C (group C) achieved a mean A1C reduction of −0.5% ± 0.03%. These findings indicate that ILI has more prominent impact on glycemic control in participants with poorly controlled diabetes and higher baseline A1C. These findings are in line with a previous study that demonstrated improvement in A1C in patients with uncontrolled diabetes after participating in an ILI.^7^ Moreover, this impact is independent of the change in body weight, since there was no difference in the magnitude of weight reduction between the three groups. Similarly, systolic BP, diastolic BP, TC, and TG decreased more prominently in participants in the higher baseline A1C groups (groups A and B) compared to the lowest baseline A1C group (group C).

To our knowledge, only two studies evaluated the relation between A1C reduction and baseline A1C in response to lifestyle modification, however the study designs and the intervention methods differ. The first study was a post‐hoc analysis, in which researchers divided the study cohort of 222 participants with T2D and obesity into three groups based on baseline A1C; group 1 with A1C ≤ 6.8%, group 2 with A1C > 6.81% and <7.7%, and group 3 with A1C ≥ 7.8%. They found that group 3 achieved the greatest improvement after 12‐weeks of lifestyle intervention.^11^ The second study was a randomized controlled 6‐month exercise intervention study of 279 participants with T2D and sedentary lifestyle.^12^ It showed that participants with baseline A1C greater than 7% achieved greater reduction in A1C than those with a baseline A1C equal or lower than 7%. In comparison to our study, the intervention methods did not include any nutritional, educational, or behavioral components. Meanwhile, that study divided participants only into two groups. It may be misrepresentative to label patients as having “inadequate metabolic control” if A1C is >7% since those patients lie within a wide A1C range of 7%–15% and their A1C reduction in response to similar amount of weight loss might be widely different. Higher baseline A1C frequently prompts physicians to start multiple anti‐hyperglycemic medications and frequently bypassing or underestimating the value of ILI in reducing A1C and may lead them to immediately start multiple, bypassing ILI.^13^

One of the advantages of this study is that we divided a large cohort of 590 participants into three groups with increasing intervals of baseline A1C: ≥6.5% to <8%, 8% to <9%, and ≥9% to provide physicians with a realistic projection of A1C reduction in response ILI, when they discuss lifestyle intervention with their patients. So far, many studies have demonstrated the importance of the magnitude of A1C reduction as prime indicator of glycemic control and its role in slowing diabetes progression, and improving cardiovascular outcomes.^8^, ^9^, ^14^

Another advantage of this study is that it was conducted among participants in ILI in real‐world clinical practice over 13 years, which may reflect a realistic expectation of such intervention. Our study results not only reinforce the role of ILI as a first‐line therapy for patients with diabetes, but also demonstrate that ILI should be also an integral component of diabetes management plan for patients with poorly controlled diabetes.

This study still has many limitations. Mainly, it is not a randomized controlled study that compare ILI to standard diabetes care. The study was also retrospective and had variability between the study groups at baseline, such as differences in percentage of participants with T1D, diabetes duration, systolic BP, diastolic BP, and percentage of participants treated with insulin. However, two key variables; body weight and BMI, were similar between the three groups at baseline. Further studies may be needed to evaluate other glycemic parameters using continuous glucose monitoring.

## 5 CONCLUSION

ILI may decrease A1C by up to 2.5% in participants with diabetes. In participants achieving similar magnitude of weight loss, A1C reduction was more prominent in those with higher baseline A1C levels. This may be valuable for clinicians to set a realistic expectation of A1C improvement in response to ILI when they discuss lifestyle modification with their patients in real‐world clinical practice.

## AUTHOR CONTRIBUTIONS

Ahmed H. Eldib collected data, conducted statistical analysis and drafted the manuscript. Shilton Dhaver collected data, edited and reviewed the manuscript. Marwa Al‐Badri edited and reviewed the manuscript. Tareq Salah edited, reviewed and prepared the manuscript for submission. Karim Kibaa edited and reviewed the manuscript. Omnia Elenani edited and reviewed the manuscript. Shaheen Tomah edited and reviewed the manuscript. Hannah Gardner edited and reviewed the manuscript. Osama Hamdy designed the study, supervised the work, edited and reviewed the manuscript.

## FUNDING INFORMATION

No funding to declare.

## CONFLICT OF INTEREST STATEMENT

OH receives research support from Novo Nordisk Inc., Eli Lilly & Company, Gilead Sciences, Inc., and the National Dairy Council. He is on the advisory board of Twin Inc, and a shareholder of Healthimation Inc. ST, AE, SD, MA, TS, KK, OE and HG have no disclosures relevant to this work.

## PRIOR PRESENTATION

Data from this study were presented at the 79th Scientific Sessions of the American Diabetes Association, June 7–11, 2019, in San Francisco, California, USA.

## Data Availability

The data contained in this manuscript are held at the Joslin Diabetes Center clinical research center.
